# Involvement of the Restoration of Cerebral Blood Flow and Maintenance of eNOS Expression in the Prophylactic Protective Effect of the Novel Ferulic Acid Derivative FAD012 against Ischemia/Reperfusion Injuries in Rats

**DOI:** 10.3390/ijms24119663

**Published:** 2023-06-02

**Authors:** Takashi Asano, Meiyan Xuan, Naohiro Iwata, Jun Takayama, Kousuke Hayashi, Yosuke Kato, Toshiya Aoyama, Hiroshi Sugo, Hirokazu Matsuzaki, Bo Yuan, Shinya Kamiuchi, Yasuhide Hibino, Takeshi Sakamoto, Mari Okazaki

**Affiliations:** 1 Laboratory of Pharmacology, Faculty of Pharmaceutical Sciences, Josai University, Saitama 350-0295, Japan; tasano@pha.u-toyama.ac.jp (T.A.); gyd2202@josai.ac.jp (H.S.); ma-tsu@josai.ac.jp (H.M.); 2 Laboratory of Organic and Medicinal Chemistry, Faculty of Pharmaceutical Sciences, Josai University, Saitama 350-0295, Japan; genbien@josai.ac.jp (M.X.); takayama@josai.ac.jp (J.T.); hayashi@azuma-g.co.jp (K.H.); yosuke_kato@nipponrika.co.jp (Y.K.); sakamoto@josai.ac.jp (T.S.); 3 Laboratory of Immunobiochemistry, Faculty of Pharmaceutical Sciences, Josai University, Saitama 350-0295, Japan; n-iwata@josai.ac.jp (N.I.); kamiuchi@josai.ac.jp (S.K.); seitaib@josai.ac.jp (Y.H.)

**Keywords:** ferulic acid derivative, middle cerebral artery occlusion, ischemia/reperfusion injury, cerebral blood flow, oxidative stress, endothelial nitrogen oxide synthetase

## Abstract

Tissue plasminogen activator, aiming to restore cerebral blood flow (CBF), has been used for acute ischemic strokes in clinics; however, its narrow therapeutic time window remains a serious concern. To develop novel prophylactic drugs to alleviate cerebral ischemia/reperfusion injuries, ferulic acid derivative 012 (FAD012) was synthesized and showed comparable antioxidant properties to ferulic acid (FA) and probably possesses the potent ability to cross the blood–brain barrier. A more potent cytoprotective effect of FAD012 against H_2_O_2_-induced cytotoxicity in PC12 cells was also observed. In vivo toxicity was not observed in rats given a long-term oral administration of FAD012, indicating its good tolerability. A one-week-course oral administration of FAD012 significantly alleviated middle cerebral artery occlusion (MCAO)-induced cerebral ischemia/reperfusion injuries in rats, accompanied by the restoration of CBF and endothelial nitrogen oxide synthetase (eNOS) expression. Treatment with FAD012 significantly restored the cell viability and eNOS expression damaged by H_2_O_2_, used to mimic MCAO-triggered oxidative stress, in rat brain microvascular endothelial cells. Our findings suggested that FAD012 protected the viability of vascular endothelium and maintained eNOS expression, ultimately contributing to the restoration of CBF, and may provide a rationale for the development of FAD012 into an effective prophylactic drug for patients at high risk of stroke.

## 1. Introduction

Acute ischemic stroke, caused by cerebrovascular thrombosis or embolism, is a serious disease with high mortality and various life-threatening sequelae such as dysphagia, hemiparesis, and higher brain dysfunction [1,2]. Thrombolytic therapy (tPA, tissue plasminogen activator) aiming to restore cerebral blood flow (CBF) has been extensively studied and widely used for acute ischemic strokes; however, its narrow therapeutic time window (within 3 to 4.5 h after the onset of symptoms) and hemorrhagic side effects remain a serious concern [3,4,5,6]. Given the difficulty of timely treatment and the scarcity of therapeutic agents for cerebral ischemia, the development of novel prophylactic drugs, which could be administered before the onset of ischemic attacks, is of critical importance and is needed urgently for populations at high risk of stroke.

It has been clarified that oxidative stress caused by reactive oxygen species (ROS) during cerebral ischemia/reperfusion and subsequent inflammatory responses play critical roles in ischemia/reperfusion injuries in the brain [7,8]. The development of novel therapeutic agents with antioxidative and anti-inflammatory activity has thus attracted considerable attention. Intriguingly, among the molecules involved in oxidative stress, nitric oxide synthase (NOS), known to catalyze L-arginine and molecular oxygen to produce nitric oxide (NO), exhibits unique features in ischemic injuries [7,8,9]. It has been demonstrated that NO synthesized by endothelial NOS (eNOS) plays an important role in promoting vasodilation, improving CBF, and ultimately protecting neurons from damage in the early stages of cerebral infarction [7,8,9]. In fact, potential neuroprotective agents, including salvianolic acid A, a caffeic acid derivative, have been shown to restore CBF associated with increases in the expression or activity of eNOS and, consequently, protect neurons from cerebral ischemic injury [10,11].

**Figure 1:**
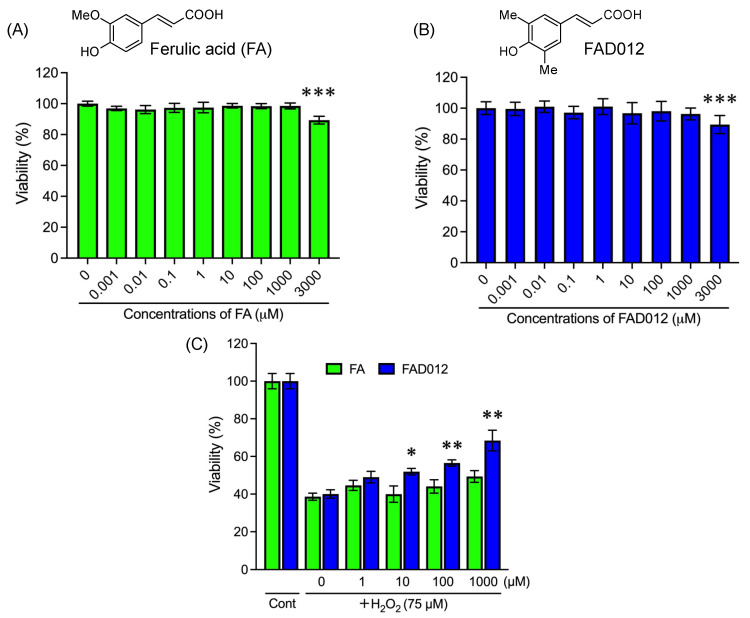
Potent cytoprotective effect of FAD012 against H_2_O_2_-induced cytotoxicity in PC12 cells in comparison with FA. Cell viability was determined via MTT assay after treatment for 24 h with either FA (**A**) or FAD012 (**B**), ranging from 0.001 to 3000 μM. ***, *p* < 0.001 vs. control. (**C**) Cell viability was measured with a CellTiter-Glo Luminescent Cell Viability assay after treatment with either FAD012 or FA, ranging from 1 to 1000 μM for 1 h prior to treatment with 75 μM of H_2_O_2_ for an additional 4 h. Relative cell viability was calculated as the ratio of the absorbance at 450 nm (MTT) or the luminescence intensity (CellTiter-Glo) of each treatment group against those of the corresponding untreated control group. *, *p* < 0.05, **, *p* < 0.01, ***, *p* < 0.001 vs. H_2_O_2_ alone. The data are represented as means ± S.E.M.; *n* = 5–7 in each group. Cont: control.

Ferulic acid (4-hydroxy-3-methoxycinnamic acid, FA) (Figure 1) is a natural phenolic compound found in products such as rice bran, coffee, and various kinds of fruits and vegetables [12]. A growing body of evidence has demonstrated that FA has the ability to suppress the progression of age-related forgetfulness and dementia [13,14,15]. Emerging evidence has also clarified that FA shows neuroprotective effects against cerebral ischemia/reperfusion-triggered injuries in vivo and in vitro [13,14,16,17,18]. The therapeutic benefits of FA are largely attributed to its potent antioxidative, anti-inflammatory, and anti-apoptotic effects [13,14,16,17,18]. Intriguingly, the maintenance of the expression of eNOS in cerebral cortex tissues has been closely related to the neuroprotective effect of FA in a rat middle cerebral artery occlusion (MCAO) model [19]. These previous findings suggested that FA might be an effective prophylactic drug for ischemic strokes. We have long been interested in the beneficial pharmacological actions of FA and demonstrated that FA suppresses the ligation of bilateral common carotid artery (2VO)-induced swallowing dysfunction in rats, in which CBF in the surficial cortex was significantly restored via treatment with FA [16]. Considering the low toxic properties and pleiotropically beneficial pharmacological actions of FA, including vasodilation [20,21], vascular endothelium protection [21,22], and anti-apoptotic action [16], we used FA as a potential leading compound to develop a series of novel ferulic acid derivatives (FADs) with potent antioxidative activity and higher lipophilicity, suggesting the usefulness of one of the FADs, FAD012 (3, 5-dimethyl-4-hydroxycinnamic acid; Figure 1) [23,24].

In the current study, we first investigated the antioxidant activity of FAD012 and FA by focusing on DPPH-scavenging activity and the inhibition of lipid peroxidation. We further compared the physicochemical properties, which are associated with the ability to cross the blood–brain barrier (BBB), between FAD012 and FA. We also compared the cytoprotective effect of FAD012 with that of FA against H_2_O_2_-induced cytotoxicity in PC12 cells. Following the confirmation of the good tolerability of FAD012, we evaluated the effects of FAD012 as well as FA on ischemia/reperfusion injuries, including neurological deficits, infarct size, and CBF using a rat MCAO model. A one-week-course oral administration of FAD012 before an MCAO operation was selected for the study based on its prophylactic protective effect against ischemia/reperfusion injuries. Given the critical role of eNOS in the restoration of CBF associated with the alleviation and/or prevention of ischemic brain injury, the alteration of eNOS expression was investigated in the cortexes of FAD012-treated rats. The in vivo effect of FAD012 on eNOS expression was further confirmed in rat brain microvascular endothelial cells (RBMVECs) treated by H_2_O_2_, which was used to mimic MCAO-triggered oxidative stress, in the absence or presence of FAD012. 

## 2. Results

### 2.1. Antioxidant and Physicochemical Properties of FAD012

As shown in Table 1, the DPPH-scavenging activity of FAD012 was in the same order of magnitude as that of FA, vitamin C (VC), and even Trolox, although the EC_50_ value of Trolox was slightly but significantly lower than those of the other three compounds (*p* < 0.05; *n* ≥ 3). A TBARS assay further demonstrated that FAD012 seemed to be more potent than FA and Trolox in inhibiting lipid peroxidation, although there was no significant difference in the IC_50_ values of each compound. VC demonstrated less antioxidant activity in the TBARS assay in comparison with the other three compounds (*p* < 0.001; *n* ≥ 3). Calculated LogP (ClogP) and polar surface area (PSA) were used to evaluate the BBB permeability, and the averages of ClogP and PSA for central nervous system (CNS) drugs were approximately 2.0 and less than 60, respectively [25,26,27]. In this regard, the values of ClogP and PSA for FAD012 were 2.44 and 57.53, respectively, in comparison with those of FA (ClogP = 1.25 and PSA = 66.76), showing that FAD012 might possess a potent ability to cross the BBB and further exert neuroprotective activity. 

### 2.2. More Potent Cytoprotective Effect of FAD012 against H_2_O_2_-Induced Cytotoxicity in PC12 Cells in Comparison to FA

FA is a well-known phenolic acid with potent antioxidative activity and has been demonstrated to have multiple protective effects on ischemic stroke [13,21]. To explore whether FAD012 is comparable to FA in terms of cytoprotective effects against oxidative stress-mediated cytotoxicity, the influence of either FAD012 or FA itself on the cell viability of PC12 was first evaluated, followed by a study on the effect of each compound against H_2_O_2_-induced cytotoxicity in the same cells. As shown in Figure 1, no cytocidal effect was observed in the cells after exposure to either FAD012 or FA, ranging from 0.001 to 1000 µM for 24 h, although a modest but statistically significant decrease in cell viability was confirmed when treated with 3000 µM of each compound, indicating their low toxicity. Next, the exposure of PC12 to 75 μM H_2_O_2_ for 4 h caused a significant decrease in cell viability (*p* < 0.001, *n* = 7). It is noteworthy that the addition of FAD012 (ranging from 1 to 1000 μM) protected the cell from the cytotoxicity of H_2_O_2_ in a dose-dependent manner. In contrast, a similar cytoprotective effect was not confirmed by the addition of FA in the same concentration range, indicating the high potency of the cytoprotective effect of FAD compared with FA. 

### 2.3. Plasma Biochemistry Analysis of Rats Given a Long-Term Administration of FAD012

Previous reports have demonstrated that FA exhibits neuroprotective effects against cerebral ischemic injuries by attenuating oxidative stress and inflammation in rodent models [17,19]. In addition, we recently demonstrated that a relatively long-term oral administration of FA (30 mg/kg, 3 weeks) alleviated chronic cerebral hypoperfusion-induced swallowing dysfunction in rats [16]. Considering the comparable antioxidant properties of FAD012 with FA and its possible BBB permeability, we thus hypothesized that the administration of FAD012 could ameliorate MCAO-induced cerebral ischemia/reperfusion injuries in rats. A plasma biochemistry analysis of rats was thus first conducted to explore its in vivo toxicity after the oral administration of either FAD012 (50 mg/kg) or 0.5% CMC (vehicle control) once a day for 10 weeks. As shown in Table 2, in comparison with the vehicle control, almost no alteration in the total plasma proteins or non-protein nitrogen substances (total protein, albumin, globulin) was observed in the FAD012-treated group. In addition, almost no alteration in the activity of alkaline phosphatase; alanine aminotransferase; amylases; and the amount of total bilirubin, glucose, creatinine, and blood urea nitrogen was observed in rats regardless of drug administration, indicating the minor effect of administering FAD012 on liver, pancreatic, and kidney functions. Electrolyte profiles further showed that the administration of FAD012 did not cause any change in the amount of sodium (Na), potassium (K), calcium (Ca), and inorganic phosphorus (IP). These results confirmed the good tolerability of FAD012 and demonstrated that the long-term administration of FAD012 has no significant in vivo toxicity in rats.

### 2.4. Improvement of Neurological Deficits Using Prophylactic Administration of FAD012

**Figure 2:**
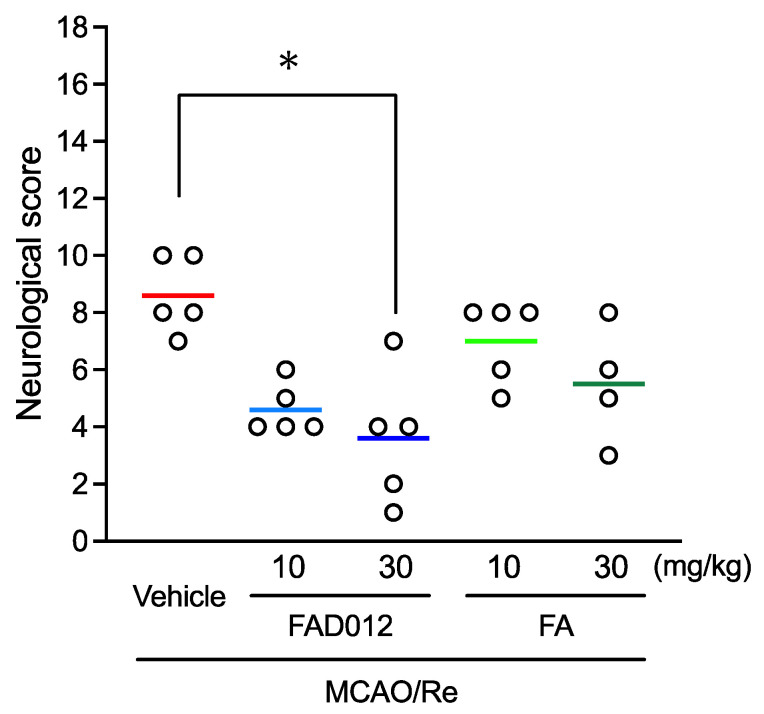
Improvement of neurological deficits using prophylactic administration of FAD012 in MCAO/Re rats. Neurological deficits were evaluated using an 18-point scale at 24 h of reperfusion after MCAO. The data are represented as means ± S.E.M.; *n* = 4–5 in each group. *, *p* < 0.05 vs. MCAO/Re-vehicle group.

MCAO/Re-induced neurological deficits were first examined. As shown in Figure 2, the neurological deficit scores were 8.6 ± 0.6 in MCAO/Re-vehicle rats. In comparison, the prophylactic administration of 10 mg/kg FAD012 decreased the neurological deficit scores from 8.6 ± 0.6 to 4.6 ± 0.4, although no significant difference was observed. Of note, the prophylactic administration of 30 mg/kg of FAD012 significantly decreased the neurological deficit scores from 8.6 ± 0.6 to 3.6 ± 1.0 (*p* < 0.05 vs. vehicle control), indicating the prophylactic protective effect of FAD012. On the other hand, no significant difference was observed between MCAO/Re-vehicle rats and MCAO/Re-FA rats, although the prophylactic administration of FA also showed a trend toward a protective effect.

### 2.5. Reduction in Infarct Size Using Prophylactic Administration of FAD012 

**Figure 3:**
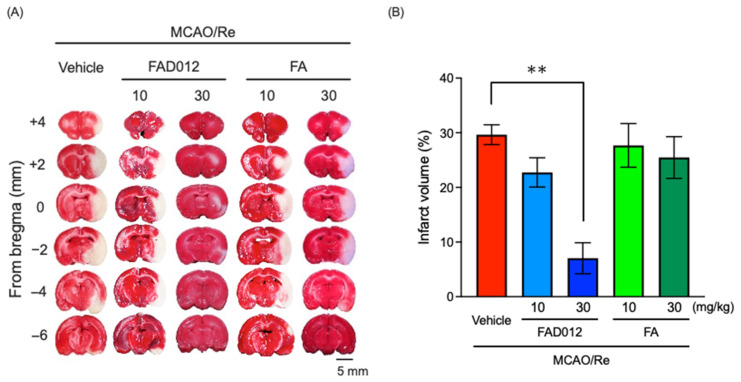
Reduction in infarct size using prophylactic administration of FAD012 in MCAO/Re rats. Representative photographs of TTC staining of coronal brain sections of each group after 24 h of reperfusion (**A**). Infarct volume in ischemic hemispheres (**B**). The data are represented as means ± S.E.M.; *n* = 4–5 in each group. **, *p* < 0.01 vs. MCAO/Re-vehicle group.

To assess acute ischemic damage in the rat brain during MCAO, the infarct size was measured using TTC staining 24 h post-reperfusion. As shown in Figure 3, an obvious white infarct area in the striatum and cortex regions was observed in the MCAO/Re-vehicle rats (29.5 ± 2.3%). The prophylactic administration of FAD dose-dependently reduced the infarct size in MCAO rats. In particular, the infarct areas in the MCAO/Re-FAD012 (30 mg/kg) rats (6.3 ± 3.5%) were significantly reduced compared with those of the MCAO/Re-vehicle rats (*p* < 0.01), reconfirming the prophylactic protective effect of FAD012. In contrast, only a modest reduction in infarct size was observed in MCAO/Re-FA rats.

### 2.6. Restoration of CBF during MCAO Using Prophylactic Administration of FAD012

**Figure 4:**
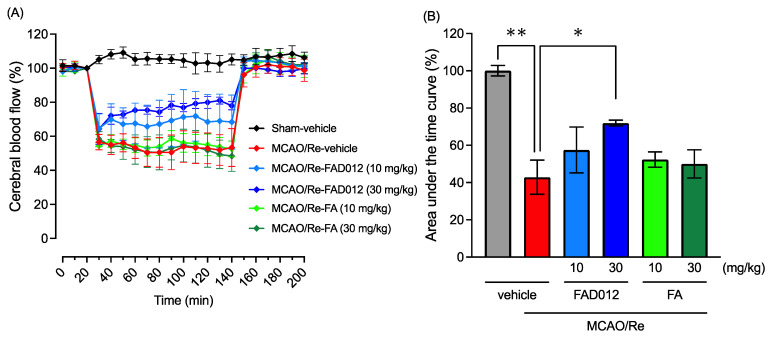
Effects of oral administration of FAD012 (30 mg/kg) on temporal changes in cerebral blood flow (CBF) in MCAO/Re rats. CBF was measured in the cerebral cortical region supplied by a middle cerebral artery using laser Doppler flowmetry. Values of CBF value (%) were calculated based on pre-MCAO (**A**). The area under the curve (AUC) for CBF was determined in each group (**B**). The data are represented as means ± S.E.M.; *n* = 4–5 in each group. **, *p* < 0.01 vs. sham-vehicle group; *, *p* < 0.05 vs. MCAO/Re-vehicle group.

The rapid restoration of blood flow in the ischemic region is considered an effective treatment strategy in ischemic strokes. To examine the prophylactic effect of FAD012 or FA against MCAO/Re-induced cerebral injury, CBF during MCAO was thus measured using laser Doppler flowmetry. As shown in Figure 4, in comparison with the sham-vehicle group, the CBF of MCAO/Re-vehicle rats remarkably dropped to 57.5 ± 5.1% at the point of MCAO onset, and the drop continued during MCAO and, furthermore, immediately recovered to 90.4 ± 4.9% after removing the suture to allow reperfusion. The assessment of the area under the curve (AUC) for CBF also confirmed the significant drop in CBF (*p* < 0.01 vs. sham-vehicle group). Of note, the remarkable drop in CBF caused by MCAO was clearly prevented by the prophylactic administration of FAD012 (10 or 30 mg/kg) but not FA, regardless of its dosage. In particular, in the MCAO/Re-FAD012 (30 mg/kg) rats, the CBF only dropped to 64.3 ± 9.1% and then steadily restored to 77.9 ± 2.4% at the end of the MCAO. In addition, the value of the AUC for the CBF of MCAO/Re-FAD012 (30 mg/kg) rats significantly increased in comparison with that of MCAO/Re-vehicle rats (*p* < 0.05). In contrast, no difference was observed in the value of the AUC for CBF between the MCAO/Re-vehicle and MCAO/Re-FA rats (10 or 30 mg/kg). Moreover, the CBF of each experimental group was almost completely restored after the onset of reperfusion. 

**Figure 5:**
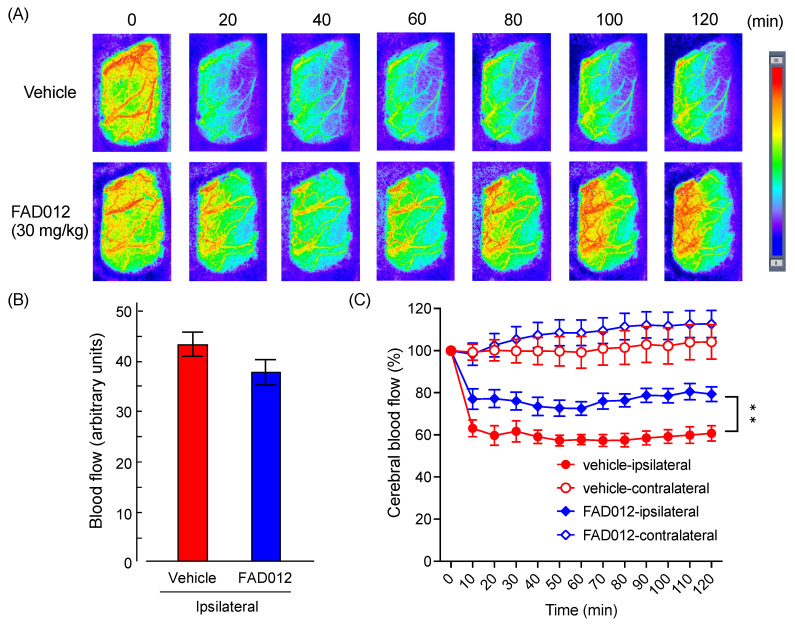
Effects of oral administration of FAD012 on temporal changes in CBF after MCAO. CBF was measured in the cerebral cortex supplied by MCA using laser Doppler flowmetry. Representative images of CBF at the indicated timepoints are pre- and post-MCAO in the vehicle and FAD012-treated groups (**A**). Arbitrary units of CBF before MCAO in vehicle- and FAD012-treated groups (**B**). Temporal changes in CBF in vehicle and FAD012-treated rats after MCAO. Values of CBF are represented relative to pre-MCAO corresponding to each group (**C**). The data are represented as means ± S.E.M.; *n* = 4–5 in each group. **, *p* < 0.01 vs. vehicle-ipsilateral.

To obtain more detailed information regarding the restoration of CBF during MCAO in MCAO/Re-FAD012 (30 mg/kg) rats, a 2D laser blood flow imager was used to monitor the real-time dynamic of CBF changes between pre- and post-MCAO in the same animal at multiple timepoints. Ischemic cortical areas (or corresponding brain areas in non-MCAO rats) were designated as regions of interest (ROIs). As shown in Figure 5, in comparison with pre-MCAO, the CBF in the ROIs decreased immediately after the MCAO operation and significantly dropped to about 60% of the baseline, and the drop continued 120 min after the onset of MCAO. Expectedly, the prophylactic administration of FAD012 significantly alleviated the drop in CBF, as evidenced by the fact that the CBF in the ROIs of the MCAO/Re-FAD012 (30 mg/kg) rats only dropped to approximately 75% of the baseline and showed a clear restoration of CBF within 120 min after MCAO onset (*p* < 0.01 vs. vehicle). In addition, almost no difference in CBF in the ROIs of the pre-MCAO rats was observed between the vehicle and FAD012 groups. Intriguingly, in comparison with the vehicle group, the prophylactic administration of FAD012 appeared to increase CBF in the ROIs in the contralateral cortex area; however, no statistical difference was observed.

### 2.7. Suppression of MCAO-Triggered Decrease in eNOS Using Prophylactic Administration of FAD012

**Figure 6:**
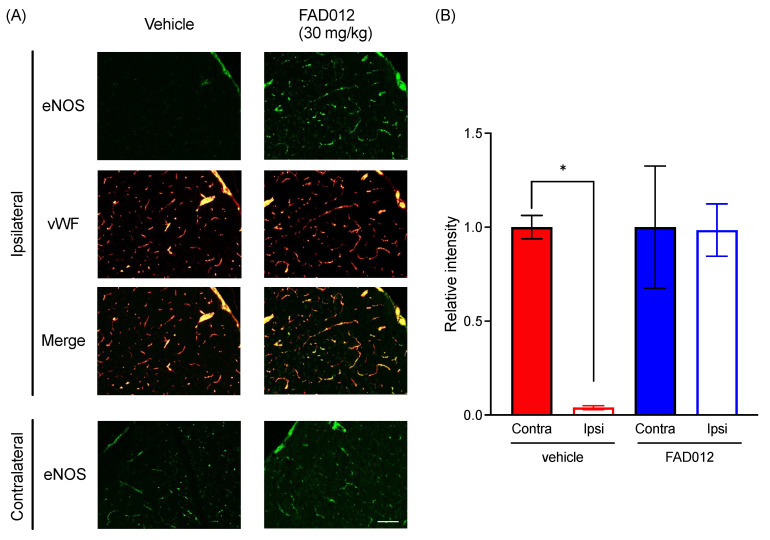
Suppression of MCAO-triggered decrease in eNOS using prophylactic administration of FAD012. Double immunostaining of endothelial nitric oxide synthase (eNOS; green) and von Willebrand factor (vWF; red) in the cortex 2 h after MCAO (**A**). Scale bar = 100 µm; fluorescence intensity of eNOS was quantified using MetaMorph Software v 7.8 focused on the relevant areas (**B**). The data are represented as means ± S.E.M.; *n* = 3 in each group. * *p* < 0.05 vs. vehicle-contralateral. Contra: contralateral; Ipsi: ipsilateral.

NO, a vasodilation gas mediator, has been reported to maintain blood flow during ischemia [28]. NO donors such as organic nitrates have also been used to treat heart failure and angina [29]. Of note, Gertz et al. demonstrated that voluntary physical activity improves long-term stroke outcomes through eNOS-dependent mechanisms related to improved angiogenesis and cerebral blood flow [30]. The expression level of the eNOS of the vascular endothelium in the cerebral cortex was thus histologically investigated. As shown in Figure 6, in comparison with the contralateral hemisphere, a remarkable decrease in the expression level of eNOS in the ipsilateral hemisphere was observed in MCAO/Re-vehicle rats 120 min post-MCAO (*p* < 0.01). However, almost no difference in the expression level of eNOS was observed between the contralateral and ipsilateral hemispheres in the MCAO/Re-FAD012 (30 mg/kg) rats, indicating that the prophylactic administration of FAD012 clearly prevented MCAO/Re from inducing the downregulation of eNOS expression.

### 2.8. Restoration of H_2_O_2_-Induced Loss of Cell Viability and eNOS Expression in RBMVEC Using FAD012

**Figure 7:**
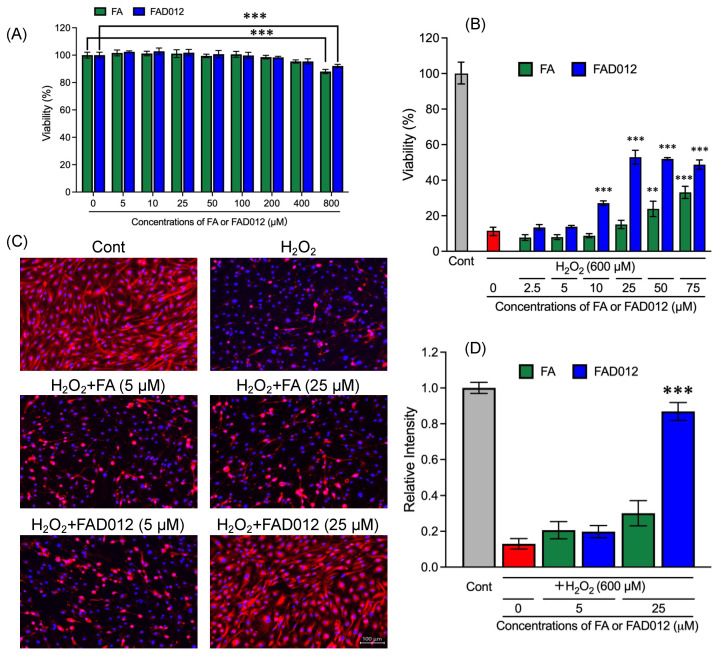
Restoration of H_2_O_2_-induced loss of cell viability and eNOS expression in RBMVEC using FAD012. Following treatment with either FAD012 or FA, ranging from 5 to 800 μM for 24 h (**A**) (***, *p* < 0.001 vs. control), and treatment with either FAD012 or FA, ranging from 2.5 to 75 μM for 1 h prior to treatment with 600 μM H_2_O_2_ for an additional 3 h (**B**) (**, *p* < 0.01, ***, *p* < 0.001 vs. H_2_O_2_ alone), cell viability was measured with a CellTiter-Glo Luminescent Cell Viability assay. Relative cell viability was calculated as the ratio of the luminescence intensity of each treatment group against those of the corresponding untreated control group. (**C**) Following treatment with 600 μM of H_2_O_2_, in the presence or absence of either FAD012 or FA for 1 h, the cells were fixed with formalin, and then, immunohistochemical staining of eNOS (Cy3 (red)) and the nucleus (DAPI (blue)) was performed, respectively, as described in “Materials and Methods”. The fluorescence intensity of eNOS and the amount of nucleus were obtained using a fluorescence microscope (BZ-X700, Keyence, Osaka, Japan). The relative intensity levels were expressed as the ratios between eNOS fluorescence intensity and the amount of nucleus and compared with those of the untreated control group (**D**) (***, *p* < 0.001 vs. H_2_O_2_ alone). The data are represented as means ± S.E.M.; *n* = 3 in each group. Cont: control.

Since our in vivo experimental data demonstrated that FAD012 exhibited prophylactic protective effects against MCAO-induced cerebral ischemia/reperfusion injuries and, coincidentally, suppressed a decrease in the expression level of the eNOS of the vascular endothelium in the cerebral cortex (Figure 3, Figure 4, Figure 5 and Figure 6 and Table 2), RBMVEC was exploited to investigate the effects of FAD012 or FA on the expression level of eNOS in cells following oxidative stress stimulation. As shown in Figure 7A, no cytocidal effect was observed following the exposure of RBMVEC to either FAD012 or FA, ranging from 5 to 400 μM for 24 h, although a modest but statistically significant decrease in cell viability was confirmed when treated with as high as 800 μM of each compound. Next, H_2_O_2_ was used to mimic MCAO-triggered oxidative stress, and our preliminary experiments showed that the optimal dose of H_2_O_2_ to induce oxidative stress was 600 µM. No cytotoxic concentrations of FAD012 and FA (ranging from 2.5 to 75 μM) were further selected to investigate their possible role in protection against H_2_O_2_-mediated cytotoxicity.

In line with our preliminary experiment, a significant decrease in cell viability was observed in RBMVEC after treatment with 600 µM of H_2_O_2_ for 3 h (*p*  <  0.001; *n* = 3) (Figure 7B). The addition of a relatively high concentration of FA (50 or 75 μM) significantly protected the cells from the cytotoxicity of H_2_O_2_. In comparison, a significant increase in cell viability was observed with the addition of FAD012 even at concentrations as low as 10 μM, indicating its superior potency against oxidative damage caused by H_2_O_2_. Immunocytochemical staining was further conducted to evaluate the status of eNOS expression in RBMVEC treated with 600 µM of H_2_O_2_ in the absence or presence of either FA or FAD012. As shown in Figure 7C,D, treatment with H_2_O_2_ caused a remarkable downregulation of eNOS expression. Intriguingly, the addition of 25 µM of FAD012, but not FA, significantly restored the expression level of eNOS (*p* < 0.001; *n* = 3), although similar restoration was not observed with the addition of a relatively low concentration of either FA or FAD012 (5 µM).

## 3. Discussion

In the present study, we demonstrated that the antioxidant properties of FAD012 are almost comparable to those of FA and Trolox in terms of DPPH-scavenging activity and the inhibition of lipid peroxidation (Table 1). An analysis of physicochemical properties showed that the values of ClogP and PSA for FAD012 were more similar to those of conventional CNS drugs [25,26,27] compared with FA, suggesting its significant ability to penetrate the BBB. We also demonstrated that, in comparison with FA, the cytoprotective effect of FAD012 against H_2_O_2_-induced cytotoxicity in PC12 cells was more potent (Figure 1). FAD012 has a chemical structure in which two methyl groups are replaced with a methoxy group and a hydrogen molecule of the aromatic ring of FA (Figure 1), and it consequently potentiates its lipophilicity and electron-donating properties. More importantly, plasma biochemistry analysis demonstrated that no significant in vivo toxicity was observed in rats given a long-term oral administration of FAD012, indicating its good tolerability and low toxicity. Given the multifaceted activity of FA in the prevention of ischemic strokes [17,19], our results thus suggested that FAD012 may serve as a promising candidate for the alleviation and/or prevention of ischemic brain injury. 

In line with our hypothesis of the neuroprotective effect of FAD012 against cerebral ischemia/reperfusion-induced injuries, the prophylactic administration of FAD012 significantly improved neurological deficits caused by MCAO/Re (Figure 2). In addition, FAD012 treatment significantly ameliorated acute ischemic damage in the rat brain as evidenced by the reduction in infarct size (Figure 3). Besides free radical scavenging, the inhibition of lipid peroxidation, and anti-inflammatory effects, FA has been shown to exert neuroprotective effects against cerebral ischemia/reperfusion-induced injuries via anti-apoptotic mechanisms in vitro and in vivo [13,18]. In this regard, we recently demonstrated that FA protected neuronal cells against 2VO-induced apoptosis by ameliorating ischemic oxidative stress [16]. Intriguingly, Cheng et al. demonstrated that the intravenous administration of FA (100 mg/kg) at the onset of MCAO significantly reduced cerebral infarct and neurological deficit scores in rats [17,31]. Similar clear neuroprotective effects for FA that were not confirmed in the current study could be explained by its low dosage (10 or 30 mg/kg) or the different administration route. In contrast, a one-week-course oral administration of FAD012 (30 mg/kg) showed an obvious neuroprotective effect, as evidenced by a significant decrease in both neurological deficit scores and infarct size (Figure 2 and Figure 3), suggesting the superior potency of the neuroprotective effects of FAD012 in comparison with FA. Even though the underlying mechanism for cellular damage from ischemia–reperfusion injuries are complex and multi-factorial, it has been clarified that oxidative stress and inflammation, two inseparable components, intersect and play a crucial role in the pathogenesis of cerebral ischemia by activating a cascade of events, and they ultimately lead to the death of neurons along with their supporting structures [7]. Targeting the two inseparable components has become an effective strategy in the prevention of ischemia–reperfusion injuries [7]. Collectively, our results suggested that the neuroprotective effects of FAD012 can be attributed to its potent activity against oxidative stress/inflammation, although the alteration in the magnitude of oxidative stress and inflammation in FAD012-treated rats obviously warrants further investigation to draw a solid conclusion.

There is no doubt that maintenance and/or rapid restoration of blood flow to the ischemic brain maximally suppresses cerebral injuries [2,6]. In the current study, two different assessment methods for CBF demonstrated that the prophylactic administration of FAD012 restored decreased CBF more efficiently in comparison with the same dosages of FA (Figure 4 and Figure 5), suggesting that the neuroprotective effects of FAD012 can be attributed to its ability to partially alleviate the drop in CBF caused by MCAO. Among the various molecules involved in the regulation of CBF, emerging evidence suggests a critical role for NO in focal cerebral ischemia by virtue of its ability to act as a primary messenger molecule mediating blood vessel relaxation, contributing to the maintenance of CBF [8,9]. It is worth noting that, as to different NOS isoforms in focal cerebral ischemia, eNOS plays a prominent role in maintaining CBF and preventing neuronal injury, while endothelial NOS (eNOS) and inducible NOS (iNOS) play key roles in neurodegeneration [8,9]. Previous studies have further demonstrated that eNOS knock-out mice exhibited more severe neurological injuries, including increased neurological deficit scores and infarct sizes after stroke than wild-type mice [32,33]. Of note, FA has been demonstrated to promote NO generation in human umbilical vein endothelial cells (HUVECs) through the PI3K/AKT/eNOS pathway [34]. More importantly, the neuroprotective effect of FA has been linked to its ability to maintain the expression of eNOS in the cerebral cortex tissues of MCAO-operated animals [19]. In the current study, a remarkable decrease in eNOS expression in MCAO/Re-vehicle rats was clearly restored using the prophylactic administration of FAD012 (Figure 6). Our in vitro experimental data further demonstrated that, in comparison with FA, the addition of FAD012 not only efficiently rescued RBMVEC from H_2_O_2_-mediated cytotoxicity but also restored the expression of eNOS in the cells (Figure 7). In fact, our research group recently revealed that a NOS inhibitor, N^G^-nitro-L-arginine methyl ester (L-NAME), abrogated the restoration of CBF via treatments with FAD012 in 2VO rats (Asano et al.; manuscript in preparation), strongly supporting the idea that FAD012 restored CBF by manipulating eNOS. Additionally, the protective effect of FA on the vascular endothelium is linked to its therapeutic benefits in vitro and in vivo [21,22]. Taken together, our findings suggested that the prophylactic administration of FAD012 protected the viability of the vascular endothelium and further maintained eNOS expression, ultimately contributing to the restoration of CBF.

In addition, FA has been reported to enhance angiogenesis in vitro and in vivo by upregulating the expression of several growth factors such as vascular endothelial growth factor (VEGF) and platelet-derived growth factor (PDGF) [22]. In this regard, we noticed that the prophylactic administration of FAD012 seemed to increase CBF in the ROIs in the contralateral cortex area after the MCAO operation; however, almost no difference in CBF in the ROIs of pre-MCAO rats was observed between the vehicle and FAD012 groups (Figure 5). Collectively, the restoration of CBF by FAD012 should be primarily attributed to its ability to maintain eNOS expression, although the possibility of the influence of FAD012 on the abovementioned growth factors could not be completely excluded. 

## 4. Materials and Methods

### 4.1. Chemicals and Reagents

FAD012 was fully characterized by 1 H and 13 C NMR, and MS spectra. The purity of FAD012 in the HPLC analysis was greater than 95%. Trolox (>99.6%), ascorbic acid (vitamin C (VC)), butylhydroxytoluene, sodium dodecyl sulfate, ethyl acetate, and DAPI were purchased from Fujifilm Wako Pure Chemical Industries, Ltd. (Osaka, Japan). 1,1-diphenyl-2-picrylhydrazyl (DPPH) was obtained from the Tokyo Chemical Industry Co., Ltd. (Tokyo, Japan). A CellTiter-Glo Luminescent Cell Viability Assay kit was purchased from Promega Corp. (Madison, WI, USA). Other reagents were obtained from Sigma-Aldrich (St. Louis, MO, USA) unless otherwise mentioned.

### 4.2. Free Radical Scavenging and Antioxidative Activities of FAD012

The free radical scavenging and antioxidative activities of FAD012 and its lead compound, FA, were assessed using DPPH and thiobarbituric acid reactive substance (TBARS) assays, respectively, as described previously [35,36]. Trolox and vitamin C (VC) were used as reference compounds. Briefly, DPPH is an organic radical with an absorption maximum of around 525 nm, and its characteristic violet color is reduced by compounds with radical scavenging activity. Compounds were diluted in ethanol at a concentration ranging from 5 µM to 1.5 mM and loaded (100 µL) onto 96-well plates. Then, 100 µL of DPPH solution (0.1 mg/mL) was added. After an incubation of 30 min at 25 °C in darkness, the absorbance of the mixture at 525 nm was measured. The efficacy of each compound was compared using the concentration needed to scavenge 50% of the radical (EC_50_). The antioxidant’s capacity to inhibit lipid peroxidation was evaluated using a TBARS assay. TBA, an index of a lipid peroxide product, is a red complex produced by a reaction with malondialdehyde that can be detected at 534 nm. In total, 20 µL of linoleic acid (5 mg/mL) and 20 µL of each compound (5–500 μM) were mixed and reacted at 80 °C for 60 min. Autoxidation was terminated with 200 µL of butylhydroxytoluene (20 μM). Then, 200 µL of 8% sodium dodecyl sulfate, 400 µL of water, and 3.2 mL of 2-thiobarbituric acid (0.67%, dissolved in 0.125 M phosphate buffer (pH 3.0)) were added, heated at 95 °C for 15 min, and then cooled on ice. In total, 4 mL of ethyl acetate was added and mixed vigorously, the mixture was centrifuged at 2000 rpm for 10 min, and the fluorescence intensity of the supernatant was measured with a fluorometer. The potency of each antioxidant was represented by IC_50_.

### 4.3. Cell Culture

PC12 (JCRB0268) cells were purchased from the Japanese Collection of Research Bioresources Cell Bank (Osaka, Japan). PC12 cells were cultured in RPMI 1640 medium (GIBCO; Thermo Fisher Scientific, Cat.11875-093, Bohemia, NY, USA) supplemented with 10% donor Horse serum (HS, SAFC Pharma, Carlsbad, CA, USA), 5% fetal bovine serum (Nichirei Biosciences Inc., Tokyo, Japan), and antibiotics (100 unit/mL penicillin, 100 μg/mL streptomycin, 0.25 μg/mL amphotericin (Wako Pure Chemical Industries, Osaka, Japan) in a humidified 5% CO_2_ atmosphere at 37 °C. The cells were seeded in a 10 cm Poly-D-Lysine-coated dish (CORNIG Inc., Corning, ME, USA) and allowed to form an adherent culture as a monolayer.

Rat brain microvascular endothelial cells (RBMVEC) were purchased from Cell Applications, Inc. (San Diego, CA, USA). RBMVEC growth medium prepared by gently mixing basal medium (Cat. R818-470) with growth supplements (Cat. R818-GS) (Cell Application, Inc., San Diego, CA, USA) was used to maintain the cell culture. The cells were cultured in monolayers in flasks (Greiner bio-one, Frickenhausen, Germany) previously coated with attachment factor solution (AFS), in accordance with the instructions of the manufacturer, in a humidified 5% CO_2_ atmosphere at 37 °C. To ensure the morphologic and phenotypic characteristics of the cells, low-passage RBMVECs (no more than 5) were used to conduct experiments.

### 4.4. Cell Viability Assay

PC12 cells were seeded in 2 × 10^4^ cells/well in Poly-D-Lysine-coated 96-well plates (CORNIG Inc., Corning, ME, USA) and cultured for 2 days. First, following treatment for 24 h with either FAD012 or FA ranging from 0.001 to 3000 μM, an MTT assay used to screen the two compounds for their respective cytotoxicity values in PC12 cells was investigated according to the method previously described with slight modifications [37]. Next, to examine the cytoprotective activity of FAD012 against oxidative stress, the cells were treated with either FAD012 or FA ranging from 1 to 1000 μM for 1 h prior to treatment with 75 μM of H_2_O_2_ for an additional 4 h. Cell viability was measured using a CellTiter-Glo Luminescent Cell Viability assay according to the method previously described with slight modifications [38].

Following treatment for 24 h with either FAD012 or FA, ranging from 5 to 800 μM, cell viability was measured using a CellTiter-Glo Luminescent Cell Viability assay according to the method previously described with slight modifications [39]. Briefly, RBMCVECs were seeded into 0.32 × 10^4^ cells/well in each well of an AFS-coated 96-well plate and cultured for 3 days, followed by exposure to various concentrations of either FAD012 or FA alone for 24 h. To examine the cytoprotective activity of FAD012 against oxidative stress, the cells were treated with either FAD or FA ranging from 2.5 to 75 μM for 1 h prior to treatment with 600 μM of H_2_O_2_ for an additional 3 h. 

### 4.5. Immunocytochemistry

The cells were cultured on an AFS-coated 8-chamber polystyrene vessel tissue-culture-treated glass slide (CORNIG Inc., Corning, ME, USA). After treatment with 600 μM H_2_O_2_, in the presence or absence of either FAD012 or FA for 1 h, cells were fixed with formalin for 10 min, treated with 0.1% Triton for 10 min, and then blocked with 5% normal goat serum (Jackson Immuno Research Laboratories, West Grove, PA, USA) for 1 h. After washing with 0.01 M PBS, the cells were reacted with a primary antibody (eNOS; 1:100, sc-376751, Santa Cruz Biotechnology, Santa Cruz, CA, USA) at 4 °C overnight. The next day, after washing with PBS, the cells were reacted with a secondary antibody (Cy3, 1:250, Invitrogen, Carlsbad, CA, USA) for 1 h at room temperature. After washing with PBS, the nuclei were stained with 1 μg/mL of DAPI reagent for 5 min and then sealed with a cover glass and observed under a fluorescence microscope (BZ-X700, Keyence, Osaka, Japan).

### 4.6. Animals

Thirty-seven adult male Sprague Dawley rats (10 weeks old) weighing 330–350 g were purchased from Japan SLC, Inc. (Hamamatsu, Japan) and housed under a temperature- (23 ± 0.5 °C) and humidity- (55 ± 10%) controlled environment with a 12/12 h light–dark cycle. The rats were provided with standard rodent chow (CE-2, CLEA Japan, Inc., Tokyo, Japan) and water ad libitum. All rats were acclimated to the environment for 1 week. FA and FAD012 were dissolved in saline containing 0.5% carboxymethyl cellulose and prepared at the time of use (Figure 1). All experiments were performed in compliance with the Guiding Principles for the Care and Use of Laboratory Animals approved by the Japanese Pharmacological Society, and the guidelines were approved by the Ethics Committee on Animal Care and Animal Experimentation at Josai University (approval number H28094, H29063, JU18060). The number of animals used was carefully estimated and kept to the minimum necessary for meaningful interpretation of the data. Animal discomfort was also minimized.

### 4.7. In Vivo Toxicity Assessment of FAD012

FAD012 was dissolved in saline containing 0.5% carboxymethyl cellulose (0.5% CMC) and prepared just before use. After acclimating for 1 week, the rats were randomly divided into two groups (*n* = 3) according to body weight using the GraphPad Prism9.5.0 software and given the following treatments: vehicle control (treated saline containing 0.5% CMC); FAD012 (50 mg/kg/day). The rats were administered p.o. as described above once a day for 10 weeks. At the end of the treatment, all animals were sacrificed and whole blood samples were collected from the inferior vena cava (IVC) of each rat. Then, following centrifugation of whole blood samples, plasma was obtained and stored at −80 °C until use. 

To evaluate the toxicity of FAD012 in vivo, a high dose (50 mg/kg) of FAD012 was orally administered to the rats (*n* = 3) for 10 weeks. The control group (*n* = 3) was treated with saline containing 0.5% carboxymethylcellulose as a vehicle. Serum samples collected from rats were used for the detection of organ toxicity using a biochemical analyzer (VetScan; ABAXIS, Union, CA, USA). Albumin, alkaline phosphatase, alanine aminotransferase, amylase, total bilirubin, and total protein as indicators of liver, gallbladder, and spleen damage were assessed by using a reagent rotor for a VetScan analyzer (Comprehensive Diagnostic Profile—Package; 89125-996, ABAXIS, Union, CA, USA). Bilirubin or creatinine, as indicators of renal impairment, were also measured.

### 4.8. Transient Middle Cerebral Artery Occlusion and Reperfusion

To examine the preventive effects of FAD012 against cerebral ischemic injuries in MCAO rats, the prophylactic administration of FAD012 to rats was conducted for 1 week prior to the MCAO procedure. In detail, after acclimating for 1 week, the rats were randomly divided into 6 groups according to body weight using the GraphPad Prism9.5.0 software: Group 1 was the sham-vehicle group (*n* = 3), orally treated with saline containing 0.5% CMC as a vehicle for 1 week and then subjected to a sham-MCAO/Re operation; Group 2 was the MCAO/Re-vehicle group (*n* = 7), orally treated with the vehicle for 1 week and then subjected to MCAO/Re; Groups 3 and 4 were the MCAO/Re-FA groups (*n* = 5, and *n* = 4), which were administered orally at 10 and 30 mg/kg FA for 1 week, respectively, and then, they were subjected to MCAO/Re; Groups 5 and 6 were the MCAO/Re-FAD groups (*n* = 4, and *n* = 8), which were administered orally at 10 and 30 mg/kg FAD for 1 week, respectively, and then, they were subjected to MCAO/Re. MCAO/Re was conducted using the method previously described with slight modification [40]. Briefly, rats were anesthetized with 2% isoflurane and maintained with 30% oxygen under spontaneous respiration. The right external carotid artery was ligated, and a 4-0 nylon monofilament with a 0.3 to 0.45 mm rounded tip was inserted into the right common carotid artery. It was threaded forward into the right internal carotid artery until the tip occluded the origin of the MCA for occlusion. After 2 h of occlusion, the filament was removed to permit reperfusion. Sham-operated rats were only ligated at the right external carotid artery. During MCAO/Re, the rectal temperature (36–38 °C) of the rats was maintained under the control of a Heating Pad System for Rodents (FHC-HPS, Muromachi Kikai Co., Ltd., Tokyo, Japan). To maintain body fluid volume, saline (1.0 mL/h) was intraperitoneally injected into the rats using a microsyringe infusion pump (KDS100: kdScientific Inc. Holliston, MA, USA).

### 4.9. Measurement of Cerebral Blood Flow by Laser Doppler Flowmetry

Cerebral blood flow (CBF) in the superficial cortical layer supplied by the right MCA was measured using laser Doppler flowmetry (ATBF-LC1: Unique Medical Co., Ltd., Tokyo, Japan) according to the method previously described with slight modifications [41]. Briefly, the probe (0A209-004, Unique Medical Co., Ltd., Tokyo, Japan) was placed on the right temporal skull surface. Measurements were continuously performed for 200 min (from 20 min before MCAO to 60 min after reperfusion) and recorded with a data acquisition system (PowerLab, ADInstruments, Spechbach, Germany). The CBF value after MCAO was expressed as a percentage of the average value for 10 min before MCAO. 

### 4.10. Measurement of Cerebral Blood Flow Using a 2D Laser Blood Flow Imager

Relative CBF was measured using a 2D laser blood flow imager (Omegazone OZ-2: Omegawave, Inc., Tokyo, Japan) according to the method previously described with minor modifications [42,43]. Briefly, under isoflurane anesthesia, rats were affixed to a brain stereotaxic frame (SR-5R-HT, Narishige, Tokyo, Japan) in a prone position, and bone fenestrations were made with a dental drill (Tas-35LX, Shofu Inc., Tokyo, Japan) on both parietal regions between coronal and lambda sutures. During the 2 h of MCAO, the CBF values of the regions were measured using a 2D laser blood flow imager. 

### 4.11. Assessment of Neurological Deficit

After 24 h of reperfusion, ischemia-induced acute symptoms were evaluated using a neurological deficit score of 18 points according to the method previously described [44]. The neurological deficits were scored by a researcher who was blinded to the experimental groups. All animals were evaluated for a maximum of 18 points: motor function testing (maximum 6 points), sensory testing (maximum 2 points), beam balance testing (maximum 6 points), and reflex and movement testing (maximum 4 points). The results were expressed as the total score of the four symptoms. 

### 4.12. TTC Staining

Rats were euthanized via decapitation under isoflurane anesthesia after 24 h of reperfusion, and their brains were removed and cut into 2 mm coronal slices using a brain matrix (World Precision Instruments, Inc., Sarasota, FL, USA). The infarct size was evaluated using TTC staining. In brief, coronal brain sections were immersed in 2% TTC at 37 °C for 6 min and were fixed with 4% paraformaldehyde at 4 °C for 24 h. Infarct areas were identified via imaging analysis software (Image J 1.52v; National Institutes of Health, Bethesda, MD, USA). The following formula determined the volume of each coronal section: V = 2/3 (area of infarction on the rostral side + area of infarction on the caudal side + √ area of infarction on the rostral side × area of infarction on the caudal side). The total volume of infarction was obtained according to the following formula: volume of infarct area (%) = [left hemisphere volume − (right hemisphere volume − the infarct volume)] × 100/left hemisphere volume.

### 4.13. Immunohistochemistry

Rats were euthanized 2 h after MCAO, and their brains were quickly removed and frozen in organic solvent (pentane: hexane, 1:2) at −100 °C using a sample freezer for cryostat (UT2000F, Leica, Bensheim, Germany). Coronal sections (10 μm thick) were cut on a cryostat (CM3050S, Leica, Bensheim, Germany). The sections were mounted on glass slides, fixed with methanol for 1 min, and washed with 0.01 M PBS. Next, the sections were preincubated with blocking reagent (Block Ace; DS Pharma Biomedical, Osaka, Japan) for 2 h and then incubated overnight at 4 °C with the primary antibodies (eNOS; 1:100; Santa Cruz Biotechnology, Santa Cruz, CA, USA and vWF; 1:200; Abcam, Burlingame, CA, USA). The sections were washed with PBS three times and then incubated with secondary antibodies (Cy3; 1:100; Chemicon International, Billerica, MA, USA, and FITC; Invitrogen, Carlsbad, CA, USA) for 2 h at room temperature. After washing with PBS, the sections were mounted on a glass slide with 80% glycerin. Each sample was observed under an upright microscope (BX53; Olympus, Tokyo, Japan). The cortex expression level of eNOS was calculated using analysis software v 7.8 (MetaMorph; Molecular Devices, San Jose, CA, USA).

### 4.14. Statistical Analysis

Statistical differences between the various groups were assessed with one-way analysis of variance (ANOVA) followed by a post hoc Tukey’s multiple comparison test. Comparisons within groups were performed with a paired *t*-test. Neurological scores were analyzed using the Kruskal-Wallis one-way analysis of variance.

## 5. Conclusions

Our results demonstrated that the prophylactic administration of FAD012 alleviated MCAO-induced cerebral ischemia/reperfusion injuries in rats. A detailed investigation into its neuroprotective effect further showed that MCAO-triggered significant decreases in CBF and eNOS were clearly restored by FAD012. We also confirmed that treatment with FAD012 significantly restored the cell viability and eNOS expression damaged by H_2_O_2_, which was used to mimic MCAO-triggered oxidative stress, in RBMVEC. Our results thus suggest that the neuroprotective effects of FAD012 against ischemia/reperfusion injuries in rats can be primarily attributed to its antioxidant activity along with the ability to maintain the eNOS expression associated with CBF restoration. Given the restriction on the use of tPA, our findings may provide a rationale for the development of FAD012 into an effective prophylactic drug for patients at high risk of stroke. However, the major limitation of our study is the lack of a bioavailability study of FAD012 in the blood and/or brains of rats, and more research is obviously needed to clarify this.

## Figures and Tables

**Table 1:** Comparison of antioxidant activities of each compound via DPPH and TBARS assay.

Compounds	DPPH Assay (EC50) (μM)	TBARS Assay (IC50) (μM)
FA	72.95 ± 5.49	34.08 ± 4.72
FAD012	72.58 ± 3.26	23.57 ± 1.85
VC	69.44 ± 4.04	1853.77 ± 88.65 ^†^
Trolox	51.87 ± 1.76 *	32.06 ± 0.95

DPPH: 1,1-diphenyl-2-picrylhydrazyl; TBARS: thiobarbituric acid reactive substances; FA: ferulic acid; FAD012: ferulic acid derivative 012; VC: vitamin C. *, *p* < 0.05 vs. FA, FAD012, and VC. †, *p* < 0.001 vs. FA, FAD012 and Trolox.

**Table 2:** Blood chemical analysis of rats with long-term high-dose oral administration of FAD012 (50 mg/kg) for 10 weeks.

	0.5% CMC (Vehicle Control)	FAD012 (50 mg/kg, p.o.)
Relative body weights (%)	156 ± 4.6	144 ± 0.6
Inspection item		
Albumin	4.8 ± 0.2	5.4 ± 0.1
Alkaline phosphatase	184 ± 14.7	294.6 ± 62.9
Alanine aminotransferase	52.5 ± 0.4	67.3 ± 21.0
Amylase	857.5 ± 75.5	992.6 ± 129.3
Total bilirubin	0.3	0.3
Blood urea nitrogen	23 ± 0.8	18 ± 0.6
Creatinine	0.55 ± 0.1	0.4 ± 0.1
Glucose	198.5 ± 11.8	154.6 ± 5.3
Total protein	7.55 ± 0.2	7.8 ± 0.4
Globulin	2.7 ± 0.1	2.4 ± 0.2
Na	141 ± 0.0	139.3 ± 1.8
K	6.05 ± 0.0	6.2 ± 0.2
Ca	10.6 ± 0.0	10.3 ± 0.1
IP	6.05 ± 0.3	6.7 ± 0.3

CMC, carboxymethyl cellulose; FAD, ferulic acid derivative; Na, sodium; K, potassium; Ca, calcium; IP, inorganic phosphorus.

## Data Availability

The datasets of the study are available from the corresponding author upon reasonable request.

## Abbreviations

AUC: area under the curve; CBF: cerebral blood flow; CMC: carboxymethyl cellulose; DPPH: 1,1-diphenyl-2-picrylhydrazyl; eNOS: endothelial nitrogen oxide synthetase; FA: ferulic acid; FAD012: ferulic acid derivative 012; MCAO: middle cerebral artery occlusion; RBMVECs: rat brain microvascular endothelial cells; Re: reperfusion; tPA: tissue plasminogen activator; TBARS: thiobarbituric acid reactive substances; VC: vitamin C; VEGF: vascular endothelial growth factor.
